# Gender differences in the prevalence of anxiety and depression and care seeking for mental health problems in Nepal: Analysis of nationally representative survey data

**DOI:** 10.1017/gmh.2024.37

**Published:** 2024-04-04

**Authors:** Md Shajedur Rahman Shawon, Fariha Binte Hossain, Moushumi Hasan, Mohammad Rifat Rahman

**Affiliations:** 1 Centre for Big Data Research in Health, University of New South Wales, Sydney, Australia; 2 School of Population Health, University of New South Wales, Sydney, Australia; 3 Independent Researcher, Dhaka, Bangladesh; 4 University of Chittagong, Chattogram, Bangladesh

**Keywords:** gender disparity, depression, anxiety, Nepal, mental health care seeking

## Abstract

**Background:**

Assessing gender disparity in mental health is crucial for targeted interventions. This study aims to quantify gender disparities in mental health burdens, specifically anxiety and depression, and related care-seeking behaviors across various sociodemographic factors in Nepal, highlighting the importance of gender-specific mental health interventions.

**Methods:**

Data from the 2022 Nepal Demographic and Health Survey was utilized, employing the Generalized Anxiety Disorder 7 scale (GAD-7) and Patient Health Questionnaire (PHQ-9) scales for anxiety and depression symptoms, respectively. Multiple logistic regression models assessed gender associations with these conditions and care-seeking behaviors.

**Results:**

Women had a higher point prevalence of anxiety (21.9% vs. 11.3%) and depression (5.4% vs. 1.7%) than men. Large variations were noted in gender disparities in the prevalence of anxiety and depression, influenced by age, geographical areas, level of education and household wealth. After adjustment for sociodemographic factors, women were more likely to experience anxiety (adjusted odds ratio (aOR) = 2.18, 95% confidence interval [CI]: 1.96–2.43) and depression (aOR = 3.21, 95% CI: 2.53–4.07). However, no difference was observed in the rates of seeking care for anxiety or depression (aOR = 1.13, 95% CI: 0.91–1.40).

**Conclusions:**

Our findings show a higher point prevalence of mental health issues among women than men, influenced by sociodemographic factors, underscoring the need for gender-focused mental health interventions in Nepal and globally.

## Impact statement

This study, encompassing over 12,000 individuals from Nepal’s latest national survey, highlights gender disparities in mental health, a crucial yet often overlooked aspect of public health. Our findings reveal stark contrasts in the prevalence of anxiety and depression between women and men, with women being almost twice as likely to experience anxiety and more than three times as likely to suffer from depression. This research not only highlights the significant gender differences in mental health issues but also underscores the complex interplay of socio-demographic factors like age, urbanicity, regional differences, education and household wealth in these disparities. Our work marks a significant step in advocating for comprehensive and gender-sensitive mental health strategies. By clearly illustrating the heightened vulnerability of women to mental health issues, and their comparable levels of care-seeking with men, the study calls for urgent attention to the unique mental health needs of different genders. This necessitates the creation of tailored policies and programs that are sensitive to the distinct challenges faced by men and women. The implications of our findings are far-reaching and global in their significance. They provide a compelling case for policymakers and public health officials to prioritize mental health services, focusing on reducing societal and structural issues that amplify gender disparities. Implementing comprehensive mental health policies and programs, particularly those that improve gender equity, reduce domestic violence and empower women, is imperative. Such initiatives could profoundly impact the reduction of mental health disparities, benefiting not only Nepal but also providing a framework for international mental health strategies. This study, therefore, represents a vital contribution to global mental health research, offering insights that could guide public health decisions and interventions across diverse populations and regions.

## Introduction

In recent times, mental disorders have been acknowledged as significant contributors to the global disease burden (Patel et al., 2018). These disorders are linked with severe morbidity, disability, reduced productivity and poor quality of life, costing the global economy US$ 1 trillion each year (World Health Organization, 2023b). The Global Burden of Diseases, Injuries and Risk Factors Study (GBD) (2019) reported that mental disorders continue to be among the top 10 leading causes of global disease burden (GBD 2019 Mental Disorders Collaborators, 2022). Globally, the age-standardized disability-adjusted life-years (DALYs) rate for mental disorders was 1,426.5 per 100,000 males and 1703.3 per 100,000 females (GBD 2019 Mental Disorders Collaborators, 2022). The intersection of demographic transitions, increased longevity, escalating non-communicable diseases (NCDs), and a high treatment gap within an overarching environment of poverty, stigma, low mental health priority and limited financing threatens to exacerbate the burden, socio-economic impact and cost of mental disorders in low- and middle-income countries (LMICs) (Patel, 2007). The *Lancet* Commission on global mental health and sustainable development underscored mental health as a fundamental human right and crucial to the development of all nations, particularly LMICs (Patel et al., 2018).

Nepal, situated between India and China in South Asia, is a diverse, landlocked country that has recently weathered political changes and the aftermath of a significant earthquake in 2015. Despite Nepal’s recent progress in socio-economic development, significant challenges persist for women in achieving political inclusion, economic security and protection against gender-based violence, all exacerbated by a deeply ingrained patriarchal system (Srivastava, 2012; Rathod et al., 2017). The country’s healthcare system is grappling with challenges related to implementing mental health services, including the rollout of community-based services, battling the stigma of mental illness, tackling financial constraints, promoting mental health legislation and efficiently utilizing human resources (Marahatta et al., 2017; Rai et al., 2021). To this day, there have been limited studies that explore the burden of mental health problems in Nepal (Upadhaya et al., 2020; Dhimal et al., 2022; Nepal WHO, 2022).

Globally and in LMICs, many studies have reported a higher prevalence of mental morbidity among women than men (Williams et al., 2008; Sagar et al., 2017; Jörns-Presentati et al., 2021; GBD, 2019 Mental Disorders Collaborators, 2022). A gender perspective allows for the identification of socio-cultural influences on women’s mental health. Evaluating gender disparities in mental health problems and care-seeking is a crucial aspect of advocating for investment in mental health, informing public health decision-making, setting priorities and accelerating the expansion of much-needed interventions.

Therefore, utilizing nationally representative data, this study aims to estimate the prevalence of anxiety and depression and care-seeking for mental health problems among adult men and women, overall and by various sociodemographic factors (i.e., age, residence, education and household wealth). Additionally, the study intends to quantify gender differences in the prevalence of anxiety, depression and care-seeking, both in the overall population and across these sociodemographic groups.

## Methods

### Datasets and study design

This cross-sectional study used secondary data from the 2022 Nepal Demographic and Health Survey (NDHS) (Ministry of Health and Population (Nepal) New ERA and ICF, 2023), which included questions related to mental health (Ewerling et al., 2020). The 2022 NDHS was executed by New ERA under the aegis of the Ministry of Health and Population, with technical assistance from ICF through the DHS Program, a United States Agency for International Development (USAID) funded project (Ministry of Health and Population (Nepal) New ERA and ICF, 2023). The 2022 NDHS received ethical approval from the Suaahara II, USAID’s integrated nutrition program. Before proceeding with the interview, each participant gave their informed written consent (Ministry of Health and Population (Nepal) New ERA and ICF, 2023). We received anonymized dataset from the DHS website (https://dhsprogram.com/data/available-datasets.cfm) after submitting a research proposal, as per their guidelines.

The 2022 NDHS utilized a sampling frame based on the updated 2011 National Population and Housing Census (NPHC) provided by the National Statistical Office (Ministry of Health and Population (Nepal) New ERA and ICF, 2023). This sampling frame encompassed all 36,020 sub-wards of Nepal, the smallest administrative units targeted in the survey. The NDHS employed a two-stage stratified sample with each of Nepal’s seven provinces divided into urban and rural strata. In the initial stage, 476 primary sampling units (PSUs) were selected in proportion to the PSU size, independent of each stratum’s sample allocation. Before the main survey, a household listing operation was conducted in all chosen PSUs, and the resultant list served as the second stage sampling frame. Thirty households were selected from each cluster, accumulating a total of 14,280 households. Sub-wards with over 300 households were segmented, and one segment was chosen proportionally to its size. Mental health surveys were conducted in half of the households (every second household). Eligible respondents included men and women aged 15–49 who were either permanent residents or visitors who stayed overnight in the selected households, resulting in a 97.4% response rate. The data collection was conducted between 5 January 2022 and 22 June 2022. Only preselected households were interviewed, with no replacements or alterations made during the survey implementation to prevent bias (Ministry of Health and Population (Nepal) New ERA and ICF, 2023).

### Mental health variables

In this study, we examined three mental health outcomes: anxiety, depression and care-seeking for mental health problems. Anxiety symptoms were assessed using the Generalized Anxiety Disorder 7 scale (GAD-7) (Spitzer et al., 2006), a validated tool for evaluating the main features of anxiety, including severe and constant worry. In addition, the GAD-7 also examines elements of common anxiety disorders such as panic disorder, social anxiety disorder and post-traumatic stress disorder. Each item in the GAD-7 was scored from 0 to 3 based on the frequency of the symptom’s occurrence in the 2 weeks preceding the survey: 0 for ‘Never’, 1 for ‘Rarely’, 2 for ‘Often’ and 3 for ‘Always’. The sum of all item scores constituted the total GAD-7 score, which could range from 0 to 21. Respondents were classified as having anxiety if their GAD-7 score was 6 or more (Spitzer et al., 2006). On the other hand, symptoms of depression were assessed using the Patient Health Questionnaire (PHQ-9), a widely accepted tool for assessing depression severity, based on the Diagnostic and Statistical Manual of Mental Disorders (DSM) criteria for depression diagnosis (Kroenke et al., 2001). The PHQ-9 items, similar to the GAD-7, are scored from 0 to 3 based on symptom frequency over the preceding 2 weeks. The scores were added to give the total PHQ-9 score (range 0 to 27), and a score of 10 or above was considered indicative of depression (Kroenke et al., 2001). Frequency distributions for GAD-7 and PHQ-10 items are given in Supplementary Tables S1 and S2. A previous study by Kroenke et al. (2001) found that a GAD-7 score of 10 offers 89% sensitivity and 82% specificity for diagnosing generalized anxiety disorder and 74% sensitivity and 81% specificity for panic disorder, while a PHQ-9 score of 10 or higher yields 88% sensitivity and specificity for major depression. Lastly, care-seeking for mental health problems was assessed among respondents identified as having either anxiety or depression by asking question on whether they sought care (yes/no) by asking the following question – “Thinking about what you yourself have experienced among the different things we have been talking about, have you ever tried to seek help?”

### Covariates

We included various sociodemographic characteristics in this study, including respondent’s age, place of residence, administrative province (Koshi, Madhesh, Bagmati, Gandaki, Lumbini, Karnali, Sudurpashchim), highest educational level and household wealth index. Age was categorized into 15–29 years, 30–39 years and 40–49 years. The definitions of rural and urban residences were guided by country-specific parameters. The socioeconomic status (SES) of the household was derived from the 2022 NDHS household wealth index, which was calculated using principal components analysis based on the quantity and variety of consumer goods they own and their housing characteristics, such as source of drinking water, toilet facilities and flooring materials. Wealth index was then assigned to each household member and used to divide the population into national wealth quintiles, each containing 20% of the population, from poorest (Q1) to richest (Q5) (Ministry of Health and Population (Nepal) New ERA and ICF, 2023).

### Statistical analysis

All analyses were conducted following the instructions in the DHS guide to analysis (the DHS Program) and used a weight variable, a stratification variable and a primary sampling using (PSU) variable in the ‘SVYSET’ programme in Stata (Statacorp, College Station, TX, USA, version 16.0) to account for the complex sampling design of survey data. All statistical tests were two-sided, and the level of significance was set at an α of 0.05.

We first looked into descriptive statistics for sociodemographic factors according to gender, using mean (standard deviation, SD) for continuous variables and proportions for categorical variables. Chi-squared test was conducted for categorical variables, whereas *t*-test was used for continuous variables. We then estimated the prevalence of anxiety and depression as well as the proportion of care-seeking among those with anxiety or depression, separately for men and women. We used sampling weights to get nationally representative prevalence estimates for each gender. We calculated 95% confidence intervals (CIs) for the prevalence estimates using a logit transform of the point estimate. We also estimated the prevalence of anxiety, depression and seeking care for mental health problems in subgroups of participants defined by age groups, area of residence, ecological region, province, education level and household’s wealth index. Next, we estimated the absolute and relative differences in the prevalence by gender (women vs. men) with corresponding 95% CIs by using two-sample tests on the equality of proportions. For estimating relative differences, we subtracted estimates for men from estimates for women and then divided that with the estimates for men.

We also conducted multiple logistic regressions yielding adjusted odds ratios (aORs) with 95% CIs to assess the associations of gender (women vs. men) with anxiety, depression and care-seeking. The regression models were adjusted for age groups, area of residence, ecological region, province, educational level and household wealth index. We also explored any further potential for effect modification by various factors for the associations of gender with anxiety, depression and care-seeking by comparing ORs across subgroups of other factors.

## Results

### Participants’ characteristics by gender

Our study included a total of 12,355 participants, consisting of 4,913 men and 7,442 women. Table 1 details the sociodemographic characteristics of all participants and further categorizes them according to gender. The majority of participants (51.8%) were in the 15–29 years age group and resided in urban areas (54.5%). Although all provinces had similar participant representation, the distribution across ecological regions was unequal, with only 8.6% from the Mountain region, 44.1% from the Hill region and 47.4% from the Terai region. About 40.9% of participants had received secondary education, and 25.9% were classified in the poorest quintile based on the household wealth index. Table 1 also reveals significant gender disparities within the sample, highlighting that it comprised 60.2% women and 39.8% men, with 27.3% of women having no education compared to 8% of men. When stratified by gender, we found a slight overrepresentation of men within the 40–49 years age group (23.1% vs. 20.7%). The distribution according to area of residence, ecological region and province remained similar between men and women. However, compared to men, fewer women had higher education (2.9% vs. 6.3%), and fewer women belonged to the richest household wealth quintile (13.8% vs. 16.3%) (Table 1).

**Table 1.** Sociodemographic characteristics of included participants from 2022 Nepal Demographic and Health Survey, overall and by gender

	Number (%)			
	Total	Men	Women	*P*-value
No. of participants	12,355 (100)	4,913 (39.8)	7,442 (60.2)	
Mean age in years (SD)	29.85 (9.97)	29.93 (10.20)	29.79 (9.82)	0.45
Age groups				0.002
15–29 years	6,406 (51.8)	2,538 (51.7)	3,868 (52.0)	
30–39 years	3,272 (26.5)	1,241 (25.3)	2,031 (27.3)	
40–49 years	2,677 (21.7)	1,134 (23.1)	1,543 (20.7)	
Area of residence				0.14
Urban	6,733 (54.5)	2,717 (55.3)	4,016 (54.0)	
Rural	5,622 (45.5)	2,196 (44.7)	3,426 (46.0)	
Ecological region				0.47
Mountain	1,058 (8.6)	407 (8.3)	651 (8.7)	
Hill	5,444 (44.1)	2,150 (43.8)	3,294 (44.3)	
Terai^*^	5,853 (47.4)	2,356 (48.0)	3,497 (47.0)	
Province				<0.001
Koshi	1,889 (15.3)	795 (16.2)	1,094 (14.7)	
Madhesh	2,144 (17.4)	882 (18.0)	1,262 (17.0)	
Bagmati	1,880 (15.2)	831 (16.9)	1,049 (14.1)	
Gandaki	1,353 (11.0)	505 (10.3)	848 (11.4)	
Lumbini	1,853 (15.0)	718 (14.6)	1,135 (15.3)	
Karnali	1,605 (13.0)	604 (12.3)	1,001 (13.5)	
Sudurpashchim	1,631 (13.2)	578 (11.8)	1,053 (14.1)	
Highest educational level				<0.001
No education	2,426 (19.6)	394 (8.0)	2,032 (27.3)	
Primary	4,347 (35.2)	1,977 (40.2)	2,370 (31.8)	
Secondary	5,059 (40.9)	2,233 (45.5)	2,826 (38.0)	
Higher	523 (4.2)	309 (6.3)	214 (2.9)	
Household’s wealth index				<0.001
Poorest	3,205 (25.9)	1,170 (23.8)	2,035 (27.3)	
Poorer	2,462 (19.9)	997 (20.3)	1,465 (19.7)	
Middle	2,430 (19.7)	965 (19.6)	1,465 (19.7)	
Richer	2,431 (19.7)	978 (19.9)	1,453 (19.5)	
Richest	1,827 (14.8)	803 (16.3)	1,024 (13.8)	

* The plains of Nepal are known as the Tarai.

### Prevalence of anxiety

The overall prevalence of anxiety was 11.3% (95% CI: 10.4%–12.2%) among men and 21.9% (95% CI: 21.0%–22.9%) among women (Table 2). For men, anxiety prevalence remained similar across all age groups. However, women aged 40–49 years exhibited a higher prevalence of anxiety (25.4%) compared to their younger counterparts (e.g., the prevalence was 21.0% among those aged 15–29 years). Men from the Mountain region reported higher anxiety levels than those from other regions, while women from the Terai region had the highest anxiety prevalence. Karnali province had the highest prevalence of anxiety for both men (17.8%) and women (27.8%). Men with higher education were more likely to experience anxiety, while women with no education were more likely to have anxiety symptoms. Households within the poorest quintile of wealth index had more individuals, both men and women, reporting anxiety symptoms.

**Table 2.** Absolute and relative gender differences in the prevalence of anxiety, overall and by sociodemographic characteristics

	Prevalence (95% CI)		Sex difference (women vs. men)	
	Men	Women	Absolute difference (95% CI)	Relative difference (95% CI)
Overall	11.3 (10.4 to 12.2)	21.9 (21.0 to 22.9)	10.6 (9.4 to 11.9)	94 (92 to 96)
Age group				
15–29 years	11.8 (10.6 to 13.1)	21.0 (19.7 to 22.3)	9.2 (7.4 to 11.0)	78 (75 to 81)
30–39 years	10.1 (8.5 to 11.9)	21.2 (19.5 to 23.0)	11.1 (8.7 to 13.5)	110 (106 to 114)
40–49 years	11.5 (9.7 to 13.5)	25.4 (23.3 to 27.7)	13.9 (11.0 to 16.8)	121 (117 to 125)
Area				
Urban	11.5 (10.5 to 12.6)	21.7 (20.6 to 22.9)	10.2 (8.6 to 11.7)	88 (86 to 91)
Rural	10.7 (9.2 to 12.4)	22.5 (20.8 to 24.2)	11.7 (9.4 to 14.1)	109 (106 to 113)
Ecological region				
Mountain	17.7 (13.4 to 22.9)	21.9 (18.2 to 26.2)	4.2 (−1.9 to 10.4)	24 (14 to 34)
Hill	11.1 (9.7 to 12.5)	19.7 (18.3 to 21.2)	8.7 (6.7 to 10.7)	78 (75 to 81)
Terai^*^	10.9 (9.7 to 12.1)	23.5 (22.2 to 24.8)	12.7 (10.9 to 14.4)	117 (114 to 119)
Province				
Koshi	13.7 (11.6 to 16.2)	24.4 (22.1 to 26.8)	10.6 (7.3 to 13.9)	77 (73 to 82)
Madhesh	6.5 (5.1 to 8.2)	22.1 (20.0 to 24.2)	15.6 (13.0 to 18.1)	240 (236 to 244)
Bagmati	13.5 (11.7 to 15.6)	19.0 (17.1 to 21.1)	5.5 (2.7 to 8.3)	41 (37 to 44)
Gandaki	8.2 (5.8 to 11.4)	17.8 (15.1 to 20.8)	9.6 (5.7 to 13.5)	117 (110 to 125)
Lumbini	11.7 (9.7 to 14.2)	21.9 (19.8 to 24.2)	10.1 (7.0 to 13.3)	86 (81 to 92)
Karnali	17.8 (13.6 to 22.9)	27.8 (23.9 to 32.1)	10.0 (3.9 to 16.2)	56 (46 to 67)
Sudurpashchim	8.5 (6.0 to 11.9)	24.2 (21.1 to 27.7)	15.8 (11.4 to 20.2)	186 (178 to 194)
Education				
No education	7.5 (5.2 to 10.5)	25.2 (23.3 to 27.2)	17.7 (14.5 to 20.9)	237 (221 to 253)
Primary	11.6 (10.2 to 13.1)	23.8 (22.1 to 25.6)	12.2 (10.0 to 14.5)	106 (103 to 108)
Secondary	11.4 (10.1 to 12.8)	19.2 (17.8 to 20.6)	7.8 (5.9 to 9.7)	68 (66 to 71)
Higher	13.2 (10.1 to 17.0)	13.3 (9.8 to 17.8)	0.1 (−5.2 to 5.3)	1 (−3 to 4)
Household wealth index				
Poorest	13.9 (11.6 to 16.6)	21.8 (19.7 to 24.1)	7.9 (4.6 to 11.2)	57 (51 to 63)
Poorer	13.4 (11.3 to 15.7)	25.5 (23.3 to 27.9)	12.2 (9.0 to 15.4)	91 (87 to 96)
Middle	8.8 (7.1 to 10.7)	24.6 (22.5 to 26.9)	15.9 (13.1 to 18.7)	181 (177 to 186)
Richer	11.0 (9.3 to 12.9)	21.3 (19.4 to 23.3)	10.3 (7.7 to 13.0)	94 (90 to 98)
Richest	10.3 (8.7 to 12.2)	16.7 (14.9 to 18.7)	6.4 (3.8 to 9.0)	62 (58 to 65)

* The plains of Nepal are known as the Tarai.

The likelihood of women experiencing anxiety was nearly double that of men, demonstrating a relative difference of 94% (Table 2). This gender disparity in anxiety (women vs. men) seemed to widen with age, increasing from 78% in the 15–29 years age group to 121% in the 40–49 years age group (Table 2). The relative gender difference (women vs. men) for anxiety was more pronounced in rural areas (109%) than in urban areas (88%). Among all provinces, Madhesh exhibited the largest gender disparity in anxiety prevalence, with a relative difference of 240%. While the prevalence of anxiety showed no gender disparity among individuals with higher education, a significant difference was observed among those without education, with a relative difference of 237%.

### Prevalence of depression

The overall prevalence of depression was 1.7% (95% CI: 1.4%–2.2%) in men and 5.4% (95% CI: 4.9%–6.0%) in women (Table 3). Depression prevalence in men was slightly lower in the 30–39 age group. Conversely, women aged 40–49 years had the highest prevalence of depression (6.2%) compared to younger women, with a 5.2% prevalence in the 15–29 age group. For men, depression prevalence was equal in both urban and rural areas. However, a higher prevalence of depression was observed among women in rural areas (6.4% vs. 5.0% in urban areas). Residents of the Mountain region, regardless of gender, reported higher depression prevalence than other regions. Karnali province had the highest depression prevalence for both men (3.6%) and women (9.3%). No definitive trend in depression prevalence was noted with education levels, though for women, there was a negative relationship observed between education and depression prevalence, with a prevalence of 6.3% among those without education and 1.6% among those with higher education. Households in the poorest wealth quintile had a greater number of individuals, men and women alike, reporting depression symptoms.

**Table 3.** Absolute and relative gender differences in the prevalence of depression, overall and by sociodemographic characteristics

	Prevalence (95% CI)		Sex difference (women vs. men)	
	Men	Women	Absolute difference (95% CI)	Relative difference (95% CI)
Overall	1.7 (1.4 to 2.2)	5.4 (4.9 to 6.0)	3.7 (3.1 to 4.3)	211 (210 to 212)
Age group				
15–29 years	2.0 (1.6 to 2.7)	5.2 (4.5 to 5.9)	3.1 (2.2 to 4.0)	154 (152 to 155)
30–39 years	1.1 (0.7 to 1.9)	5.4 (4.5 to 6.4)	4.2 (3.1 to 5.4)	375 (373 to 377)
40–49 years	1.8 (1.2 to 2.8)	6.2 (5.1 to 7.6)	4.4 (3.0 to 5.9)	247 (245 to 249)
Area				
Urban	1.7 (1.3 to 2.2)	5.0 (4.4 to 5.6)	3.2 (2.5 to 4.0)	189 (188 to 190)
Rural	1.8 (1.2 to 2.6)	6.4 (5.5 to 7.5)	4.6 (3.4 to 5.8)	254 (252 to 256)
Ecological region				
Mountain	2.9 (1.4 to 5.9)	7.2 (5.1 to 10.2)	4.3 (1.1 to 7.6)	148 (143 to 153)
Hill	1.6 (1.2 to 2.3)	5.0 (4.2 to 5.8)	3.3 (2.3 to 4.3)	202 (200 to 203)
Terai	1.7 (1.3 to 2.3)	5.6 (4.9 to 6.3)	3.9 (3.0 to 4.7)	226 (224 to 227)
Province				
Koshi	2.3 (1.5 to 3.6)	6.5 (5.2 to 8.0)	4.2 (2.5 to 5.9)	179 (177 to 181)
Madhesh	0.5 (0.2 to 1.2)	5.0 (4.0 to 6.2)	4.5 (3.3 to 5.7)	929 (928 to 931)
Bagmati	1.9 (1.3 to 2.8)	4.4 (3.4 to 5.5)	2.5 (1.2 to 3.8)	131 (129 to 132)
Gandaki	1.6 (0.7 to 3.4)	4.0 (2.8 to 5.7)	2.4 (0.5 to 4.3)	153 (150 to 157)
Lumbini	2.1 (1.3 to 3.3)	4.9 (3.9 to 6.2)	2.9 (1.4 to 4.4)	139 (137 to 142)
Karnali	3.6 (1.9 to 6.7)	9.3 (7.0 to 12.4)	5.7 (2.3 to 9.2)	159 (153 to 165)
Sudurpashchim	1.5 (0.6 to 3.4)	6.8 (5.1 to 9.0)	5.3 (3.0 to 7.6)	357 (353 to 361)
Education				
No education	1.1 (0.4 to 2.8)	6.3 (5.3 to 7.5)	5.2 (3.7 to 6.7)	473 (466 to 481)
Primary	2.2 (1.6 to 2.9)	6.5 (5.6 to 7.6)	4.3 (3.1 to 5.5)	198 (197 to 200)
Secondary	1.5 (1.1 to 2.2)	4.4 (3.7 to 5.2)	2.9 (2.0 to 3.8)	184 (183 to 186)
Higher	1.4 (0.6 to 3.3)	1.6 (0.6 to 4.0)	0.2 (−1.7 to 2.1)	12 (10 to 13)
Household wealth index				
Poorest	3.0 (2.0 to 4.5)	6.4 (5.2 to 7.9)	3.4 (1.6 to 5.2)	115 (112 to 118)
Poorer	1.8 (1.1 to 2.8)	6.3 (5.1 to 7.7)	4.6 (3.0 to 6.1)	260 (258 to 263)
Middle	1.2 (0.7 to 2.2)	6.4 (5.3 to 7.7)	5.2 (3.7 to 6.6)	419 (416 to 421)
Richer	1.8 (1.2 to 2.8)	4.9 (3.9 to 6.0)	3.1 (1.8 to 4.4)	171 (169 to 173)
Richest	1.3 (0.8 to 2.2)	3.4 (2.6 to 4.5)	2.1 (0.9 to 3.2)	159 (157 to 160)

Overall, women were over twice as likely to suffer from depression compared to men, demonstrating a relative difference of 211%. This gender disparity in depression was highest (375%) in the 30–39 age group. Moreover, the relative difference between women’s and men’s depression prevalence was more pronounced in rural areas (254%) compared to urban areas (189%). Among all provinces, Madhesh showed the largest gender disparity in depression prevalence, with a relative difference of 929%. Education level played a significant role in the gender disparity in depression prevalence, with a 473% difference among those without education, but no difference among those with higher education. The largest gender disparity in depression was observed in women from households in the middle wealth quintile compared to men.

### Care-seeking for mental health problems

Overall, a higher proportion of women sought care for anxiety or depression (34.1%, 95% CI: 31.8%–36.4%) compared to men (29.3%, 95% CI: 25.7%–33.2%) (Table 4). Younger men were more likely to seek care than older men, but such difference by age was not observed among women. While we observed no urban vs. rural differences in care-seeking among men and women, there were wide variations in care-seeking across provinces, particularly among men. For women, care-seeking for mental health problems varied from 32.8% among those with no education to 36.0% among those with secondary education. However, for men, it varied widely between 12.0% in those with no education and 34.2% among those with secondary education. For both sexes, care-seeking did not vary significantly according to household wealth index (Table 4).

**Table 4.** Absolute and relative gender differences in care-seeking for anxiety or depression, overall and by sociodemographic characteristics

	Prevalence (95% CI)		Sex difference (women vs. men)	
	Men	Women	Absolute difference (95% CI)	Relative difference (95% CI)
Overall	29.3 (25.7 to 33.2)	34.1 (31.8 to 36.4)	4.8 (0.4 to 9.2)	16 (4 to 29)
Age group				
15–29 years	34.3 (29.2 to 39.8)	35.3 (32.1 to 38.6)	1.0 (−5.2 to 7.2)	3 (−14 to 20)
30–39 years	25.6 (18.7 to 33.9)	32.1 (27.9 to 36.6)	6.5 (−2.2 to 15.2)	26 (−5 to 56)
40–49 years	21.0 (14.8 to 28.9)	33.7 (29.1 to 38.5)	12.7 (4.2 to 21.1)	60 (35 to 86)
Area				
Urban	29.1 (24.9 to 33.7)	34.2 (31.5 to 37.1)	5.1 (−0.1 to 10.3)	18 (3 to 32)
Rural	29.7 (23.1 to 37.3)	33.7 (29.9 to 37.8)	4.0 (−4.1 to 12.2)	14 (−14 to 41)
Ecological region				
Mountain	32.1 (19.9 to 47.4)	26.9 (18.7 to 37.0)	−5.3 (−21.7 to 11.1)	−16 (−50 to 17)
Hill	30.7 (24.9 to 37.1)	31.6 (27.9 to 35.5)	0.9 (−6.2 to 8.1)	3 (−16 to 22)
Terai	27.8 (23.0 to 33.2)	36.2 (33.2 to 39.2)	8.4 (2.5 to 14.3)	30 (11 to 50)
Province				
Koshi	25.5 (18.5 to 34.1)	32.7 (27.7 to 38.2)	7.2 (−2.1 to 16.5)	28 (5 to 51)
Madhesh	18.8 (11.0 to 30.3)	39.0 (34.0 to 44.3)	20.2 (9.4 to 31.0)	107 (52 to 163)
Bagmati	25.6 (19.5 to 32.8)	32.2 (27.1 to 37.8)	6.6 (−1.9 to 15.2)	26 (11 to 41)
Gandaki	18.9 (8.6 to 36.6)	33.8 (26.0 to 42.6)	14.9 (−0.9 to 30.6)	79 (19 to 138)
Lumbini	40.7 (31.4 to 50.8)	34.3 (29.2 to 39.9)	−6.4 (−17.5 to 4.7)	−16 (−50 to 19)
Karnali	41.6 (28.4 to 56.1)	31.8 (24.3 to 40.4)	−9.8 (−25.8 to 6.3)	−23 (−67 to 20)
Sudurpashchim	41.9 (25.3 to 60.6)	30.7 (24.0 to 38.4)	−11.2 (−30.3 to 7.9)	−27 (−127 to 74)
Education				
No education	12.0 (4.1 to 30.4)	32.8 (28.8 to 37.1)	20.8 (8.2 to 33.3)	173 (−40 to 385)
Primary	25.9 (20.6 to 32.0)	33.1 (29.3 to 37.2)	7.2 (0.3 to 14.2)	28 (11 to 45)
Secondary	34.2 (28.7 to 40.2)	36.0 (32.1 to 40.0)	1.7 (−5.3 to 8.7)	5 (−10 to 21)
Higher	29.0 (17.9 to 43.4)	34.7 (20.9 to 51.7)	5.7 (−14.2 to 25.5)	20 (5 to 34)
Household wealth index				
Poorest	29.3 (21.4 to 38.8)	25.8 (21.1 to 31.0)	−3.6 (−13.5 to 6.4)	−12 (−41 to 16)
Poorer	31.6 (24.1 to 40.3)	36.0 (31.2 to 41.2)	4.4 (−5.1 to 13.9)	14 (−13 to 40)
Middle	32.2 (23.2 to 42.9)	31.9 (27.3 to 36.8)	−0.4 (−11.3 to 10.5)	−1 (−48 to 46)
Richer	19.7 (13.7 to 27.6)	41.2 (36.3 to 46.4)	21.5 (12.9 to 30.1)	109 (84 to 134)
Richest	34.7 (26.6 to 43.9)	34.0 (28.4 to 40.1)	−0.7 (−11.1 to 9.6)	−2 (−25 to 20)

Women were slightly more likely to seek mental health care than men, showing a relative difference of 16%. A much larger relative difference between women and men in care-seeking behavior was observed for those aged 40–49 years and living in Madhesh province. We did not observe substantial sex differences in care-seeking according to quintiles of household wealth index except for those in the richer (Q4) quintile.

### Association between gender and mental health outcomes

**Figure 1.**
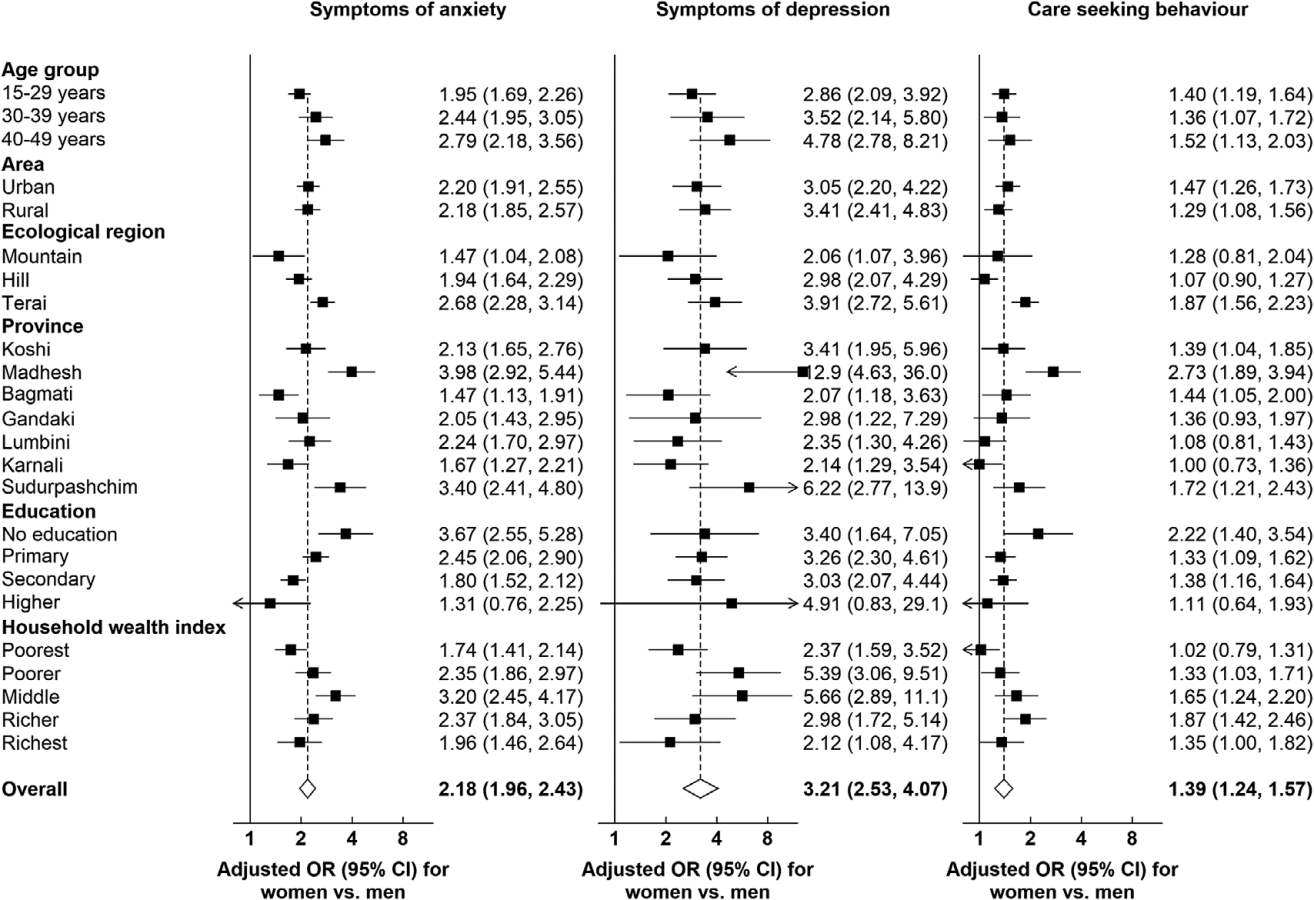
Associations of gender (women vs. men) with prevalence of anxiety, depression and care-seeking for mental health problems. Multiple logistic regressions were adjusted for age, province, area of residence, education level and household wealth index, as appropriate. Odds ratios (ORs) are represented by squares, and their corresponding 95% CIs are represented by lines.

Figure 1 illustrates the overall relationships between gender and anxiety, depression, as well as care-seeking, and their variations across different sociodemographic factors. The overall adjusted odds ratio (aOR) for gender (women vs. men) was 2.18 (95% CI: 1.96–2.43) for anxiety, 3.21 (95% CI: 2.53–4.07) for depression and 1.13 (95% CI: 0.91–1.40) for care-seeking. When analyzed according to different sociodemographic factors, stronger associations between gender and anxiety were observed in individuals aged 40–49 years, those residing in the Terai region, inhabitants of the Madhesh and Sudurpashchim provinces, individuals with no education, and those belonging to the middle quintile of the household wealth index. As for the relationship between gender and depression, a significantly stronger association was detected only in the Madhesh province. Regarding the relationship between gender and care-seeking for mental health, significant associations were evident for individuals in the Terai region, Madhesh province and those from richer (Q4) households.

## Discussion

This study, involving 12,355 individuals from the most recent nationally representative sample in Nepal, has identified several key findings related to gender differences in the prevalence of anxiety, depression and care-seeking for mental health symptoms. Women were found to be nearly twice as likely to have anxiety (21.9% vs. 11.3%) and more than three times as likely to have depression (5.4% vs. 1.7%) compared to men. Even though the prevalence of anxiety and depression varied significantly across other sociodemographic factors, these variations manifested differently for men and women. We found large variations in gender disparities in the prevalence of anxiety and depression by other sociodemographic factors, including age groups, urbanicity, region, province, educational attainment and household wealth index. Although women were far more likely to suffer from anxiety and depression than men, the care-seeking for anxiety or depression among women was comparable to men.

Our findings align with previous research suggesting that women are more likely to experience anxiety and depression than men. For instance, the National Mental Health Survey in Nepal, conducted in 2019–2020, found that 5.1% of women aged 18 and above were currently suffering from mental disorders, such as schizophrenia, bipolar disorder, major depressive disorder, or suicidal thoughts, compared to 3.4% men suffering from mental disorders (Dhimal et al., 2022). The 2019 GBD estimates highlighted that women had a higher prevalence of major depressive disorder compared to men (4.3% vs. 2.8%) in Nepal (Nepal WHO, 2022). Studies in different contexts have also demonstrated higher rates of mental disorders among women. The World Health Organization (WHO) reports that depression is approximately 50% more prevalent among women than men (World Health Organization, 2023a). Anxiety disorders are also significantly more common in women throughout their lifespans than in men (Farhane-Medina et al., 2022). A recent scoping review on the burden of psychiatric disorders in Africa showed that major depression and generalized anxiety disorders were more common among women than men (Greene et al., 2021). The Adult Psychiatric Morbidity Survey from England revealed that one in five women (20.7%) and one in eight men (13.2%) suffered from a common mental disorder, which included generalized anxiety disorder, depression, phobias, obsessive-compulsive disorder, panic disorder and unspecified disorders (Gulland, 2016). However, not all research showed a higher mental health burden among women. For example, the National Mental Health Survey (NMHS) of India reported the current prevalence of ‘any mental morbidity’ was estimated at 7.5% for women, lower than the 13.9% reported for men (Gautham et al., 2020).

Various social and gender-specific factors are thought to contribute to the higher prevalence of mental disorders among women, particularly in low- and middle-income countries (LMICs) like Nepal. Social factors such as poverty, urbanization, internal migration and lifestyle changes often play a role in the high prevalence of mental illnesses (Rathod et al., 2017). Similarly, in our study, we found that women who had no education and were from poorest households had the highest burden of depression and anxiety. In addition, issues like depression, anxiety, psychological distress and experiences of domestic violence disproportionately affect women in different countries and cultures (Srivastava, 2012). The convergence of multiple roles that women play, gender discrimination and factors such as domestic violence and sexual abuse are believed to contribute to women’s mental health issues (Srivastava, 2012; Rathod et al., 2017). A significant indicator of gender disadvantage is the experience of intimate partner violence. There is robust evidence linking domestic violence to detrimental effects on women’s mental health in both affluent and poorer countries (Coker et al., 2002; Ogbe et al., 2020). A population-based survey from India highlighted the strong association between sexual violence within marriage, a subject often considered taboo and depression in women (Patel et al., 2006). Additionally, experiences associated with gender disadvantage, such as marrying during adolescence or being widowed or separated, were also identified as risk factors (Patel et al., 2006). Societies in many LMICs, particularly in South Asia, often exhibit a dominant patriarchal structure (Niaz and Hassan, 2006). Additionally, the unique physiological and behavioral demands of pregnancy and motherhood, with special emphasis on depression during the peripartum period, can also contribute to the increased mental health burden on women (Eid et al., 2019).

Our study emphasized that various socio-demographic factors, including age groups, area of residence, education level and household wealth index, considerably influence the gender disparity in mental health issues. Notably, the gender disparity for anxiety and depression was significantly higher in rural areas compared to urban areas. This disparity may be indicative of fewer available facilities for women as well as shortage of trained mental health professionals, and lower levels of mental health literacy. Furthermore, cultural factors and stigmas associated with mental health can potentially impede the seeking of care among women, particularly in more traditional, rural societies (Rathod et al., 2017). Additionally, we observed a significantly higher gender disparity in the burden of anxiety and depression among those with no education, indicating women with lower level of education may face more mental health stressors than men. A recent study conducted in India with a representative population revealed that mental health problems were more prevalent among middle-aged individuals, residents of urban-metro areas, less educated individuals and households with lower income (Evans-Lacko et al., 2018).

Our study also highlighted that although women suffer significantly higher burden of mental health problems than men, their care-seeking behavior was similar to men (aOR 1.13, 95% CI: 0.91–1.40). Because gender also influences the amount of control individuals have over the determinants of their health, including their economic status, social standing and access to resources and treatment in society (Patel, 2007), there is a critical need for comprehensive, accessible mental health services for women across different demographic and socioeconomic strata to mitigate these disparities.

Our study has several strengths, including employing standardized methodologies and well-established instruments, specifically the GAD-7 (Spitzer et al., 2006) and PHQ-10 (Kroenke et al., 2001), to identify those with anxiety and depression. Moreover, we analyzed data from a nationally representative survey using validated questionnaires to assess mental health issues and care-seeking behaviors. However, our study also has certain limitations. Despite their robustness, neither GAD-7 nor PHQ-10 fully covers all facets of anxiety and depression. For instance, using a threshold score of 10, GAD-7 has an 89% sensitivity and 82% specificity for generalized anxiety disorder, and a 74% sensitivity and 81% specificity for panic disorder (Kroenke et al., 2007). Meanwhile, a PHQ-9 score of 10 or higher indicates a sensitivity and specificity of 88% each for major depression (Kroenke et al., 2001). This implies potential misses in identifying some individuals with anxiety or depression, which must be kept in mind when interpreting the results. The 2022 NDHS, conducted amidst the COVID-19 pandemic, necessitates a careful consideration of the pandemic’s impact on the study’s findings and the observed gender disparities. Moreover, the NDHS lacks information on non-binary gender concepts and future research should focus on mental health within this population in Nepal. Another limitation of the study is its lack of detailed information on the types of treatment and service providers, as well as potential differences in service preferences between males and females, which could have been valuable to local stakeholders. Finally, the cross-sectional survey design of the study restricts the ability to infer causality.

In conclusion, our study highlights significant gender disparities in the prevalence of anxiety and depression in Nepal. Women were more likely to suffer from these mental health disorders but slightly more likely to seek care compared to men. These disparities were found to vary significantly based on factors such as age, urbanicity, regional and provincial differences, educational attainment and household wealth index. These findings emphasize the need for tailored and gender-sensitive mental health strategies to address the unique needs and circumstances faced by men and women. Policymakers should prioritize mental health services by addressing the underlying societal and structural issues that contribute to gender disparities. Comprehensive and context-specific mental health policies and programs aiming to improve gender equity, reduce domestic violence and empower women could have a significant impact on reducing mental health disparities.

## Supporting information

Shawon et al. supplementary material Shawon et al. supplementary material

## Data Availability

The 2022 Nepal Demographic and Health Survey dataset used in this study is publicly available at this link: https://dhsprogram.com/data/.

## Supplementary material

The supplementary material for this article can be found at http://doi.org/10.1017/gmh.2024.37.
