# Supplementary material for: Gender differences in the prevalence of anxiety and depression and care seeking for mental health problems in Nepal: Analysis of nationally representative survey data
Source: Glob Ment Health (Camb). 2024 Apr 4;11:e46. doi: 10.1017/gmh.2024.37 (PMC11058515; doi:10.1017/gmh.2024.37)
Supplement: Shawon et al. supplementary material [file S2054425124000372sup001.docx]

**Gender differences in the prevalence of anxiety and depression and care seeking for mental health problems in Nepal: analysis of nationally representative survey data**

**Supplementary materials**

| **Supplementary table 1: Distributions of symptoms of anxiety and depression in the 2 weeks preceding among men** | | | | |
| --- | --- | --- | --- | --- |
|  | **N (%)** | | | |
|  | **Never** | **Rarely** | **Often** | **Always** |
| Symptoms of anxiety |  |  |  |  |
| Feeling nervous, anxious, or on edge | 3081 (62.7) | 1509 (30.7) | 255 (5.2) | 68 (1.4) |
| Not being able to stop or control worrying | 4318 (87.9) | 505 (10.3) | 75 (1.5) | 15 (0.3) |
| Worrying too much about different things | 3138 (63.9) | 1438 (29.3) | 270 (5.5) | 67 (1.4) |
| Trouble relaxing | 3931 (80.0) | 798 (16.2) | 153 (3.1) | 31 (0.6) |
| Being so restless that it is hard to sit still | 4108 (83.6) | 646 (13.1) | 122 (2.5) | 36 (0.7) |
| Becoming easily annoyed or irritable | 2957 (60.2) | 1624 (33.1) | 244 (5.0) | 87 (1.8) |
| Feeling afraid as if something awful might happen | 3704 (75.4) | 1005 (20.5) | 155 (3.2) | 49 (1.0) |
|  |  |  |  |  |
| Symptom of depression |  |  |  |  |
| Little interest or pleasure in doing things | 3783 (77.0) | 963 (19.6) | 135 (2.7) | 31 (0.6) |
| Feeling down, depressed, or hopeless | 3837 (78.1) | 915 (18.6) | 134 (2.7) | 27 (0.5) |
| Trouble falling asleep or staying asleep or sleeping too much | 3790 (77.1) | 931 (18.9) | 156 (3.2) | 35 (0.7) |
| Feeling tired or having little energy | 3832 (78.0) | 959 (19.5) | 108 (2.2) | 13 (0.3) |
| Poor appetite or overeating | 4114 (83.7) | 684 (13.9) | 95 (1.9) | 18 (0.4) |
| Feeling bad about yourself or that you are a failure or have let yourself or your family down | 4318 (87.9) | 482 (9.8) | 87 (1.8) | 23 (0.5) |
| Trouble concentrating on things such as reading the newspaper or watching television | 4222 (85.9) | 605 (12.3) | 75 (1.5) | 9 (0.2) |
| Moving or speaking so slowly that other people could have noticed or the opposite | 4562 (92.9) | 297 (6.0) | 39 (0.8) | 12 (0.2) |
| Thoughts that you would be better off dead or of hurting yourself in some way | 4800 (97.7) | 95 (1.9) | 14 (0.3) | 3 (0.1) |
|  |  |  |  |  |

| **Supplementary table 2: Distributions of symptoms of anxiety and depression in the 2 weeks preceding among women** | | | | |
| --- | --- | --- | --- | --- |
|  | **N (%)** | | | |
|  | **Never** | **Rarely** | **Often** | **Always** |
| Symptoms of anxiety |  |  |  |  |
| Feeling nervous, anxious, or on edge | 3789 (50.9) | 2707 (36.4) | 671 (9.0) | 275 (3.7) |
| Not being able to stop or control worrying | 5737 (77.1) | 1246 (16.7) | 346 (4.6) | 113 (1.5) |
| Worrying too much about different things | 4073 (54.7) | 2505 (33.7) | 659 (8.9) | 204 (2.7) |
| Trouble relaxing | 5160 (69.3) | 1732 (23.3) | 408 (5.5) | 141 (1.9) |
| Being so restless that it is hard to sit still | 5809 (78.1) | 1223 (16.4) | 319 (4.3) | 91 (1.2) |
| Becoming easily annoyed or irritable | 3289 (44.2) | 3147 (42.3) | 792 (10.6) | 213 (2.9) |
| Feeling afraid as if something awful might happen | 4932 (66.3) | 1942 (26.1) | 458 (6.2) | 109 (1.5) |
|  |  |  |  |  |
| Symptom of depression |  |  |  |  |
| Little interest or pleasure in doing things | 5326 (71.6) | 1564 (21.0) | 436 (5.9) | 115 (1.5) |
| Feeling down, depressed, or hopeless | 4876 (65.5) | 1958 (26.3) | 485 (6.5) | 123 (1.7) |
| Trouble falling asleep or staying asleep or sleeping too much | 5126 (68.9) | 1758 (23.6) | 429 (5.8) | 129 (1.7) |
| Feeling tired or having little energy | 4659 (62.6) | 2154 (28.9) | 508 (6.8) | 121 (1.6) |
| Poor appetite or overeating | 5317 (71.4) | 1715 (23.0) | 336 (4.5) | 74 (1.0) |
| Feeling bad about yourself or that you are a failure or have let yourself or your family down | 6502 (87.4) | 702 (9.4) | 187 (2.5) | 51 (0.7) |
| Trouble concentrating on things such as reading the newspaper or watching television | 5920 (79.5) | 1212 (16.3) | 256 (3.4) | 54 (0.7) |
| Moving or speaking so slowly that other people could have noticed or the opposite | 6619 (88.9) | 647 (8.7) | 144 (1.9) | 32 (0.4) |
| Thoughts that you would be better off dead or of hurting yourself in some way | 6895 (92.6) | 423 (5.7) | 94 (1.3) | 30 (0.4) |
|  |  |  |  |  |
