# Supplementary material for: Gender differences in the prevalence of anxiety and depression and care seeking for mental health problems in Nepal: Analysis of nationally representative survey data
Source: Glob Ment Health (Camb). 2024 Apr 4;11:e46. doi: 10.1017/gmh.2024.37 (PMC11058515; doi:10.1017/gmh.2024.37)
Supplement: Peer review history

# Editor report: Recommendation: Gender differences in the prevalence of anxiety and depression and care seeking for mental health problems in Nepal: Analysis of nationally representative survey data — R0/PR2

**DOI:** 10.1017/gmh.2024.37.pr2

**Affiliations:** School of Public Health and Preventive Medicine, Monash University Faculty of Medicine Nursing and Health Sciences, Australia

No accompanying comment.

# Editor report: Decision: Gender differences in the prevalence of anxiety and depression and care seeking for mental health problems in Nepal: Analysis of nationally representative survey data — R0/PR3

**DOI:** 10.1017/gmh.2024.37.pr3

**Affiliations:** London School of Hygiene & Tropical Medicine, United Kingdom of Great Britain and Northern Ireland

No accompanying comment.

# Editor report: Recommendation: Gender differences in the prevalence of anxiety and depression and care seeking for mental health problems in Nepal: Analysis of nationally representative survey data — R1/PR5

**DOI:** 10.1017/gmh.2024.37.pr5

**Affiliations:** School of Public Health and Preventive Medicine, Monash University Faculty of Medicine Nursing and Health Sciences, Australia

Thank you for submitting the revised paper, which I am pleased to accept subject to providing evidence that the original study had received ethics approval.

# Editor report: Decision: Gender differences in the prevalence of anxiety and depression and care seeking for mental health problems in Nepal: Analysis of nationally representative survey data — R1/PR6

**DOI:** 10.1017/gmh.2024.37.pr6

**Affiliations:** London School of Hygiene & Tropical Medicine, United Kingdom of Great Britain and Northern Ireland

No accompanying comment.
